# Association between psoas muscle mass index and bone mineral density in patients undergoing hemodialysis

**DOI:** 10.1038/s41598-024-84873-4

**Published:** 2025-01-02

**Authors:** Kiyonori Ito, Susumu Ookawara, Hidenori Sanayama, Hideo Kakuda, Chieko Kanai, Katsuo Iguchi, Mitsutoshi Shindo, Keisuke Tanno, Shun Ishibashi, Masafumi Kakei, Kaoru Tabei, Yoshiyuki Morishita

**Affiliations:** 1 Division of Nephrology, First Department of Integrated Medicine, Saitama Medical Center, Jichi Medical University, 1-847 Amanuma-cho, Omiya-ku, Saitama City, 330-8503 Saitama Japan; 2 Division of General Medicine, First Department of Integrated Medicine, Saitama Medical Center, Jichi Medical University, Saitama, Japan; 3 Department of Clinical Radiology, Minami-uonuma City Hospital, Niigata, Japan; 4 Department of Dialysis, Minami-uonuma City Hospital, Niigata, Japan; 5 Department of Internal Medicine, Glicina Clinic Shonandai, Fujisawa City, Kanagawa Japan; 6 Division of Radiology, First Department of Integrated Medicine, Saitama Medical Center, Jichi Medical University, Saitama, Japan; 7 Division of Cardiovascular Medicine, First Department of Integrated Medicine, Saitama Medical Center, Jichi Medical University, Saitama, Japan; 8 Department of Internal Medicine, Minami-uonuma City Hospital, Niigata, Japan

**Keywords:** Psoas muscle mass index, Bone mineral density, Hemodialysis, Computed tomography, Sarcopenia, Osteoporosis, Diseases, Medical research, Nephrology, Risk factors, Engineering

## Abstract

Patients undergoing dialysis are at risk of osteoporosis and sarcopenia because of mineral and bone disorders or malnutrition. Additionally, maintaining muscle mass is important to prevent osteoporosis. The psoas muscle mass index (PMI) was recently used to evaluate muscle mass. However, few studies have evaluated the association between the PMI and bone mineral density (BMD); therefore, we examined the association between PMI and BMD in the femoral neck (FN) of 80 patients (45 males, age, 71 (60–76) years; dialysis duration, 74 (36–140) months) undergoing hemodialysis. FN-BMD was measured using dual-energy X-ray absorptiometry, and PMI was evaluated using psoas muscle areas on computed tomography. FN-BMD and PMI were significantly higher in males than in females. In a correlation analysis, sex, BMI, serum creatinine levels, HbA1c levels, and PMI were positively correlated with FN-BMD, whereas age, history of bone fracture, difficulty in walking and bone-specific alkaline phosphatase level were negatively correlated. In the multivariate regression analysis using clinical factors significantly correlated to FN-BMD, including PMI, both sex (standardized coefficient: 0.249, *p* = 0.028) and PMI (standardized coefficient: 0.249, *p* = 0.038) were extracted. Multivariable linear regression analysis using PMI and traditional osteoporosis factors revealed that PMI was significantly and independently associated with FN-BMD (standardized coefficient: 0.308, *p* = 0.010). In conclusion, PMI was positively associated with FN-BMD. Attention should be paid to the possibility of decreased BMD with decreased muscle mass.

## Introduction

Decreased skeletal muscle mass and osteoporosis are significant medical and social problems affecting the elderly population, associated with increased mortality and hospitalizations^1,2^. Patients with chronic kidney disease (CKD) including those undergoing dialysis could also face heightened concerns, because the prevalence of sarcopenia increases with CKD stage progression^3^. Additionally, CKD-mineral and bone disorder (MBD) could affect bone condition. As the average age continues to increase in both dialysis and dialysis-initiated patients^4^, various concerns about muscle and bone disorders would grow.

Recently, as one of the evaluated methods about muscle mass, the psoas muscle mass index (PMI) has been used in various clinical settings^5–7^. Additionally, PMI was reportedly evaluated in patients with CKD, including those undergoing hemodialysis (HD)^8–12^. Psoas muscle mass is measured by using bilateral psoas muscle area at the 3rd lumber spine (L3) level on computed tomography (CT) scans, and there are some opportunities that CT scans are performed for investigating malignancy including renal carcinoma in dialyzed patients. Although muscle mass cannot be evaluated in medical institutions without muscle measuring equipment, i.e. bioelectrical impedance analysis (BIA), PMI measured by CT might provide new information about muscle status. Actually, PMI is strongly correlated with the skeletal muscle mass index (SMI) in patients undergoing HD^7^.

Bone mineral density (BMD) is a method used to evaluate bone quantity, and its measurement has been recommended for patients with CKD, including those undergoing HD^13^. Although CKD-related bone disorders are extremely complex, BMD measurement may be useful for understanding bone condition^13,14^. In the general elderly population, SMI was positively corelated with femoral neck (FN)-BMD^15^, and the results were similar to those in patients undergoing HD^16^. Regarding the association between BMD and PMI, there are several reports on patients not undergoing dialysis^17–19^, however, thus far, the association in patients undergoing HD is uncertain. Therefore, we aimed to investigate the association between BMD and PMI calculated using CT imaging in patients undergoing HD.

## Materials and methods

This retrospective study included patients who had undergone intermittent HD and were older than 20 years. Each patient underwent maintenance HD three times per week for a duration of 3–5 h per session. We investigated patients undergoing HD who met the following criteria: (i) underwent abdomen-pelvic CT evaluation and (ii) underwent FN-BMD evaluation. These evaluations were performed as an annual screening check within one month, and we excluded patients who were not undergo these checks within one month. The study was approved by the Institutional Review Board of Minami-Uonuma City Hospital (R3-3), Japan, and conformed to the provisions of the Declaration of Helsinki (revised in Tokyo in 2004). Due to the retrospective nature of the study, the Institutional Review Board of Minami-Uonuma City Hospital waived the need to obtain informed consent.

### Data collection

Baseline patient characteristics and other relevant data, including primary disease of CKD, past medical history, and medication, were collected from the medical records of our hospital. Blood pressure and pulse rate were measured in the supine position before HD. Body mass index (BMI) was calculated using the dry weight. Blood samples were obtained from arteriovenous fistulas before HD. Difficulty in walking was observed by the medical staff before HD.

## Measurement of BMD

Dual energy X-ray absorptiometry (Horizon A, HOLOGIC Japan, Tokyo, Japan) was used to evaluate BMD in theFN. To minimize variations in BMD measurements, a single radiologic technologist performed the scanning and BMD data calculations for all HD patients. In addition to measuring the absolute values of FN-BMD, the T-scores and standard deviations (SD) compared with healthy young sex-matched controls were automatically calculated. Patients were categorized into three groups of patients with normal BMD (T-score > -1SD), patients with decreased BMD (-1 SD < T-score < -2.5 SD), and patients with osteoporosis (T-score < -2.5 SD) respectively,

in accordance with the definition of the World Health Organization^20^. Based on these results, we determined the prevalence of osteoporosis in this study.

## Measurement of PMI

The PMI was measured using a multi-detector row CT scanner (Aquilion 64; Toshiba Medical Systems, Tochigi, Japan) and Ziostation (Ziosoft Inc, Tokyo, Japan). Evaluation by CT was performed annually to check for acquired cystic disease of the kidneys and renal carcinoma in patients undergoing HD. Using these imaging techniques, the psoas muscle was evaluated at the L3 level to measure the cross-sectional areas of the bilateral psoas muscles, as previously reported^6–11^. In this study, psoas muscle areas were measured by a specific radiologist using the manual trace method. Based on these results, the PMI was calculated by normalizing the cross-sectional areas for height (cm^2^/m^2^).

### Statistical analysis

Data are expressed as mean ± SD or median and interquartile range. We first determined whether the data were normally distributed using the Shapiro–Wilk test to evaluate each variable. The correlation between FN-BMD and clinical parameters was evaluated using Pearson’s correlation or Spearman’s rank correlation for normally and non-normally distributed data, respectively. Multivariate linear regression analysis was performed to identify the independent factors for FN-BMD in patients undergoing HD. All analyses were performed using SPSS Statistics for Windows version 25.0 (IBM, Armonk, NY, USA). Statistical significance was set at *p* < 0.05.

## Results

**Fig. 1:**
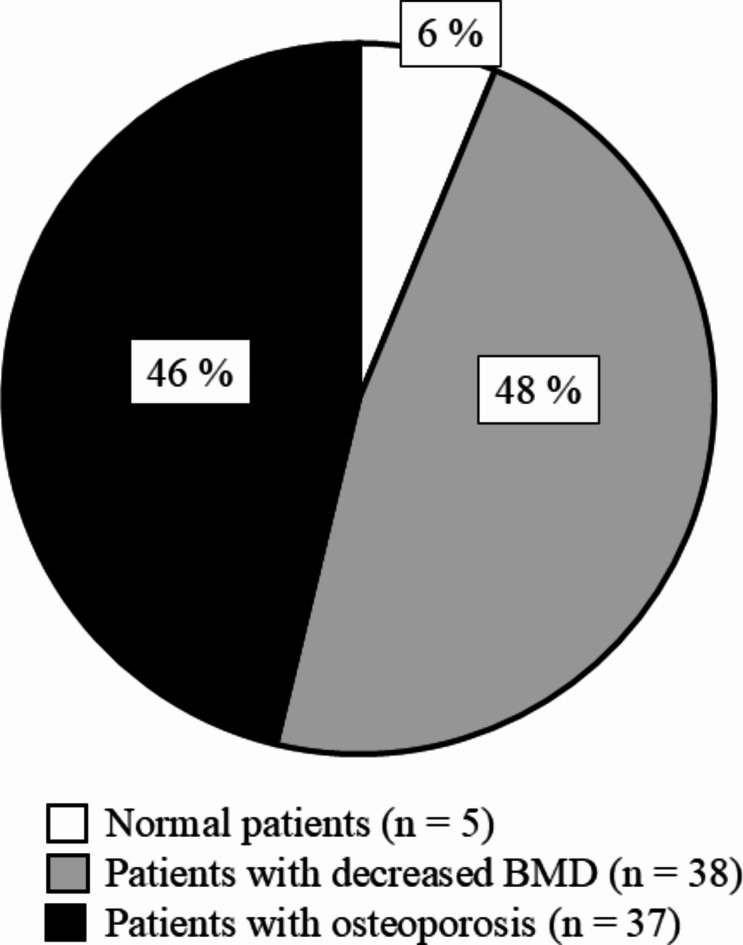
The percentages of osteoporosis in patients undergoing HD. The white space indicates the percentage of normal patients. The gray space shows the percentage of patients with decreased FN-BMD. The black space indicates the percentage of patients with osteoporosis.

The patients’ general characteristics are summarized in Table 1. Eighty HD patients (45 males and 35 females; age, 71 (60–76) years; HD duration, 74 (36–140) months) were included. The mean FN-BMD of all included HD patients was 0.55 ± 0.11 g/cm^2^, and the mean FN-BMD of male patients was significantly higher than that of female patients (male vs. female: 0.59 ± 0.10 g/cm^2^ vs. 0.49 ± 0.12 g/cm^2^). Furthermore, FN-BMD was also evaluated using T-score, and only 5 patients had normal BMD, while 38 patients had decreased BMD and 37 patients had osteoporosis, as shown in Fig. 1. Additionally, the mean PMI of all included HD patients was 4.7 ± 1.3 cm^2^/m^2^, and the mean PMI of male patients was significantly higher than that of female patients (male vs. female: 5.30 ± 1.25 cm^2^/m^2^ vs. 3.85 ± 1.00 cm^2^/m^2^).

**Table 1:** Patient characteristics and correlation between femoral neck BMD and clinical parameters.

*n* = 80	mean (median)	*r*	*p*
Sex Male, n (%)	45 (56)	0.417^#^	< 0.001
Age, years	71 (60–76)	-0.274^#^	0.014
HD duration, months	74 (36–140)	-0.111^#^	0.325
Past medical history and physical status, n (%)			
Diabetes mellitus	29 (36)	0.006^#^	0.960
Bone fracture	24 (30)	-0.312^#^	0.005
Difficulty of walking	18 (23)	-0.368^#^	0.001
Vital sign and physical findings			
Systolic BP, mmHg	156 ± 22	-0.007	0.952
Diastolic BP, mmHg	81 ± 15	0.127	0.263
Heart rate, /min	72 ± 12	0.123	0.276
Body Mass Index, kg/m^2^	22.5 ± 3.9	0.448	< 0.001
Medication, n (%)			
Vitamin D analog	62 (78)	0.126^#^	0.266
Phosphate binder	63 (79)	-0.175^#^	0.120
Calcimimetics	31 (39)	0.027^#^	0.814
Laboratory findings			
Albumin, g/dL	3.5 (3.3–3.8)	0.139^#^	0.219
Serum sodium, mEq/L	138 (136–140)	-0.017^#^	0.881
Serum potassium, mEq/L	5.0 ± 0.7	-0.141	0.214
Serum calcium, mg/dL	8.6 ± 0.7	-0.158	0.163
Serum phosphate, mg/dL	5.1 (4.4–6.1)	0.148^#^	0.190
BUN, mg/dL	63 ± 15	0.132	0.244
Cr, mg/dL	10.6 ± 3.0	0.508	< 0.001
Hb, g/dL	11.6 (10.9–12.0)	0.059^#^	0.601
TSAT, %	29.0 (20.7–35.9)	-0.023^#^	0.842
Ferritin, ng/mL	131 (59–236)	-0.026^#^	0.818
Total cholesterol, mg/dL	155 (130–183)	0.053^#^	0.642
HDL cholesterol, mg/dL	50 ± 14	-0.102	0.368
Triglyceride, mg/dL	99 (68–151)	0.171^#^	0.129
HbA1c, %	5.3 (4.9–5.8)	0.251^#^	0.025
β2-microglobulin, mg/L	27.9 ± 5.7	0.015	0.895
Intact PTH, pg/mL	139 (98–190)	0.108^#^	0.339
ALP, U/L	252 (189–317)	-0.060^#^	0.600
BAP, µg/L	13.0 (10.6–15.8)	-0.320^#^	0.004
TRACP-5b, mU/dL	416 (294–656)	-0.206^#^	0.068
Psoas Muscle Mass Index, cm^2^/m^2^	4.7 ± 1.3	0.504	< 0.001
Femoral neck BMD, g/cm^2^	0.55 ± 0.11	-	-

Abbreviations: BMD, bone mineral density; HD, hemodialysis; BP, blood pressure; BUN, blood urea nitrogen; Cr, creatinine; Hb, hemoglobin; TSAT, transferrin saturation; HDL, high density lipoprotein; PTH, parathyroid hormone; ALP, alkaline phosphatase; BAP, bone-specific alkaline phosphatase; TRACP-5b, tartrate-resistant acid phosphatase 5b.

Categorical data are presented as number (%), while continuous data are presented as mean ± standard deviation or median (interquartile range).

^#^Spearman’s rank correlation for skewed distributed data.

**Fig. 2:**
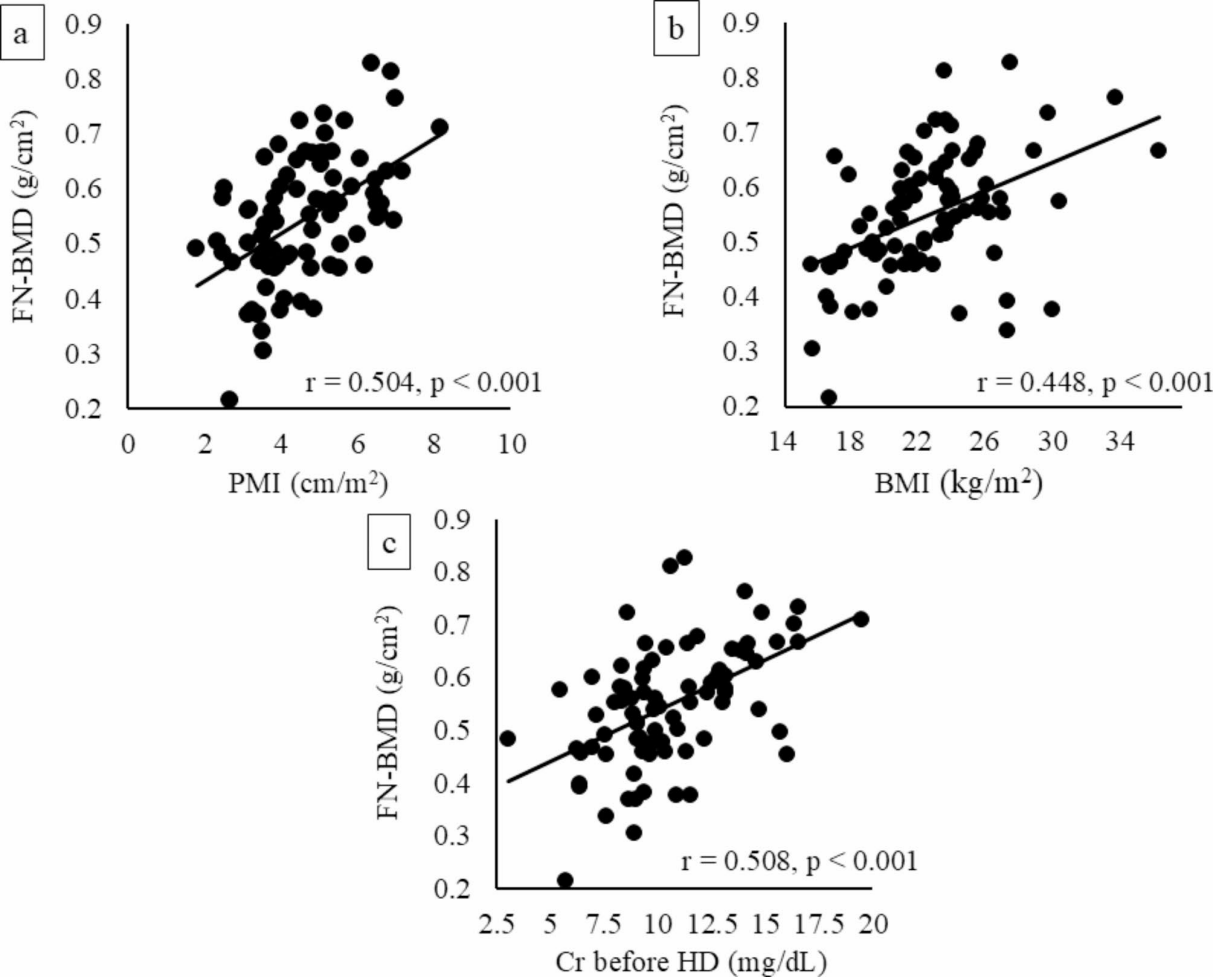
Correlations between FN-BMD and clinical parameters which could reflect body trunk in patients undergoing HD. (**a**) Correlations between FN-BMD and PMI. (**b**) Correlations between FN-BMD and BMI. (**c**) Correlations between FN-BMD and serum Cr before HD.

We examined the correlation between FN-BMD and the clinical parameters presented in Table 1. FN-BMD showed significant positive correlations with sex (*r* = 0.417, *p* < 0.001), BMI (*r* = 0.448, *p* < 0.001), serum creatinine (Cr) (*r* = 0.508, *p* < 0.001), HbA1c (*r* = 0.251, *p* = 0.025) and PMI (*r* = 0.504, *p* < 0.001), while FN-BMD showed significant negative correlations with age (*r* = -0.274, *p* = 0.014), history of bone fracture (*r* = -0.312, *p* = 0.005), present difficulty of walking (*r* = -0.368, *p* = 0.001) and bone specific alkaline phosphatase (ALP) (*r* = -0.320, *p* = 0.004). As shown in Fig. 2, clinical parameters that reflect the body size in patients undergoing HD, that is, BMI, serum Cr levels before HD and PMI^16^, were strongly correlated with FN-BMD.

In the multivariate linear regression analysis, we analyzed three models using factors that reflect physical size in patients undergoing HD; BMI, serum Cr and PMI, in addition to other significant factors. Multivariate linear regression analysis was performed as shown in Table 2. In model 1 using PMI, sex (standardized coefficient: 0.249, *p* = 0.028) and PMI (standardized coefficient: 0.249, *p* = 0.038) were extracted. In model 2 using BMI, sex (standardized coefficient: 0.415, *p* < 0.001) and BMI (standardized coefficient: 0.364, *p* < 0.001) were extracted. In model 3 using serum Cr, sex (standardized coefficient: 0.286, *p* = 0.006) and serum Cr (standardized coefficient: 0.287, *p* = 0.022) were extracted. Furthermore, to elucidate the relationship between FN-BMD, PMI, and traditional osteoporosis risk factors, including calcium (Ca), phosphate, and parathyroid hormone (PTH), we conducted a detailed investigation using multivariable linear regression analysis (Table 3). In model 1, sex (standardized coefficient: 0.248, *p* = 0.025), bone fractures (standardized coefficient: -0.268, *p* = 0.005), and PMI (standardized coefficient: 0.299, *p* = 0.010) were significantly and independently associated with FN-BMD. In model 2, which incorporated traditional osteoporosis risk factors into model 1, no changes were observed in the associations between FN-BMD and the factors that were significantly correlated in model 1, particularly PMI.

**Table 2:** Multivariable linear regression analysis for femoral neck-BMD in patients undergoing HD.

	Coefficient	95%CI	Standardized coefficient	*p* value
**Model 1**				
Age	–0.001	–0.003 − 0.001	–0.132	0.191
Sex	0.057	0.006–0.107	0.249	0.028
Bone fracture	–0.046	–0.098 − 0.006	–0.176	0.082
Difficulty of walking	–0.018	–0.086 − 0.050	–0.067	0.601
BAP	–0.003	–0.007–0.000	–0.186	0.079
HbA1c	0.003	–0.028–0.034	0.020	0.846
PMI	0.021	0.001–0.041	0.249	0.038
**Model 2**				
Age	–0.001	–0.003 − 0.001	–0.133	0.150
Sex	0.094	0.054–0.134	0.415	< 0.001
Bone fracture	–0.048	–0.097 − 0.001	–0.194	0.056
Difficulty of walking	–0.025	–0.087 − 0.038	–0.092	0.432
BAP	–0.002	–0.006 − 0.001	–0.129	0.197
HbA1c	–0.017	–0.047 − 0.014	–0.106	0.282
BMI	0.011	0.005–0.017	0.364	< 0.001
**Model 3**				
Age	0.000	–0.003 − 0.002	–0.041	0.716
Sex	0.065	0.020–0.111	0.286	0.006
Bone fracture	–0.048	–0.100 - − 0.004	–0.195	0.070
Difficulty of walking	–0.022	–0.089 − 0.045	–0.082	0.516
BAP	–0.002	–0.006 − 0.001	–0.137	0.198
HbA1c	0.001	–0.030–0.032	0.006	0.952
Cr	0.011	0.002–0.020	0.287	0.022

Abbreviations: BMD, bone mineral density; HD, hemodialysis; PMI, psoas muscle mass index; BMI, body mass index; BAP, bone-specific alkaline phosphatase; Cr, creatinine; CI, confidence interval.

**Table 3:** Multivariable linear regression analysis of the relationship among femoral neck-BMD, PMI, and traditional osteoporosis risk factors in patients undergoing HD.

	Coefficient	95%CI	Standardized coefficient	*p* value
**Model 1**				
Age	–0.001	–0.003 − 0.001	–0.127	0.187
Sex	0.057	0.007–0.108	0.248	0.025
Bone fracture	–0.067	–0.113 − 0.021	–0.268	0.005
PMI	0.026	0.006–0.045	0.299	0.010
**Model 2**				
Age	–0.001	–0.003 − 0.001	–0.100	0.322
Sex	0.053	0.001–0.104	0.227	0.044
Bone fracture	–0.069	–0.116 - − 0.022	–0.275	0.004
PMI	0.026	0.006–0.046	0.308	0.010
Ca	–0.016	–0.049 − 0.016	–0.092	0.323
P	0.008	–0.006–0.022	0.106	0.271
Intact PTH	–2.365 × 10^–5^	0.000–0.000	–0.017	0.858

Abbreviations: BMD, bone mineral density; PMI, psoas muscle mass index; HD, hemodialysis; CI, confidence interval; Ca, calcium; P, phosphate; PTH, parathyroid hormone.

## Discussion

In this study, we investigated the association between FN-BMD and PMI in patients undergoing HD, and evaluated whether the psoas muscle, which supports the hip, including the femur, could be associated with FN-BMD. There are several reports on these associations in patients without CKD^17–19^; however, to our knowledge, there are no reports on the association between PMI and FN-BMD in patients undergoing HD.

Many patients undergoing HD have experienced bone fractures. In this study, almost all study patients had osteopenia or osteoporosis, and the Dialysis Outcomes and Practice Patterns Study reported that FN bone fracture rates in HD patients were higher in any countries than those in non-dialysis patients^21^. In Japan, the standard incidence ratios in FN bone fractures in HD patients are approximately five-times higher than those in general population^22^. After bone fracture in patients undergoing HD, the 1-year mortality was more than 10% and survival rates without hospitalization were only 40% in Japanese patients^21^; therefore, we need to pay attention to the management of bone fracture risk. Especially, CKD-MBD management including serum Ca, phosphate and PTH was needed for preventing bone fracture^13,14^. In a recent observational study, hyperphosphatemia and high PTH levels was associated with increased fragility fractures^23^. Furthermore, intact PTH levels reflect bone histology in patients undergoing HD^24^, and such secondary hyperparathyroidism would lead to increased bone resorption and formation^14^. As the results, decreased bone mass and increased bone fragility can cause bone fracture. In addition, ALP or serum magnesium (Mg) might be another factor related FN fracture in dialyzed patients. High ALP and low Mg levels are associated with a risk of new-onset hip fracture^25,26^. Thus, although various factors could be related to bone condition or fractures in patients undergoing HD, we focused on muscle mass including PMI. Recently, osteosarcopenia, a novel concept describing the relationship between osteoporosis and sarcopenia, could be highlighted in various clinical settings^27–29^. Among elderly populations, patients with osteosarcopenia have demonstrated poorer prognosis concerning both fracture risk and mortality compared to those without the condition^27^. In addition, patients with CKD, including HD, had a higher prevalence of osteosarcopenia with reduced survival rates^28,29^. Furthermore, as loss of muscle mass itself could contribute to falls or bone fractures^30^, it is important to clarify the association between bone condition and muscle status, including PMI.

PMI is a recently developed method for evaluating muscle mass. In previous studies, the PMI was strongly correlated with SMI in the general population^5^ and in patients undergoing HD^16^; therefore, the PMI reflects whole body muscle mass. In contrast, the psoas muscle also anatomically supports the pelvis or femur and contributes to the maintenance of good posture or walking stability. As the result, weakness of psoas muscle might affect falls or physical activity. In patients with CKD in Japan, the prevalence of sarcopenia increases with CKD progression^31^, and the cumulative incidence of falls also increases with CKD progression^32^. Thus, although it is important to maintain muscle mass, including that of the psoas muscle, to prevent falls and maintaining physical activity, skeletal muscle cannot often be evaluated without equipping dual-energy X-ray absorptiometry or BIA in clinical settings. Even in such situations, PMI evaluations might contribute to diagnosing decreased muscle mass or sarcopenia in various patients^6,7,11,12^.

In this study, FN-BMD was also associated with Cr levels before HD, and actually, it could be independently associated factors with muscle mass in patients undergoing HD^9,33^. Because serum Cr is a metabolic product derived from muscle and Cr itself usually accumulates in the serum of patients undergoing HD, Cr before HD would reflect muscle mass status in addition to uremic toxins.

As the associated mechanisms between BMD and the muscle mass, mechanical stimuli, including bone loading or physical activity, and nutritional deficiency might be raised. In a clinical study on postmenopausal elderly women, whole-body muscle stimuli were associated with increased BMD^34^. Furthermore, in recent basic research, the mechanical load by BW on bone tissues contributes to increased bone quantity via periosteal osteocrin, which could facilitate bone formation^35^. Thus, these bone or muscle stimuli could have a positive influence on BMD, and large body size, i.e. higher BMI or higher PMI, might lead to a larger mechanical load on bone or muscle. Additionally, physical activity was also associated with BMD. Elderly patients with higher BMD reportedly have longer active time, shorter sedentary time and more daily steps^36^. In contrast, patients undergoing HD may take fewer daily steps, especially on the day of HD therapy^37^. Furthermore, in patients with Parkinson’s disease, freezing of gait, which leads to a reduction in physical activity and muscle mass, has been linked to lower FN-BMD^38^. Additionally, in younger patients with anorexia nervosa who participated in an exercise program, the duration of weight-bearing exercise was positively associated with both higher BMD and increased lean body mass, while lower BMI was inversely related to BMD^39^. These findings suggest that mechanical stimuli for bone and muscle, elicited through physical activity, may play an important role in preserving both BMD and muscle mass, although obesity or worsening of metabolic status by an increase in BW could cause a decrease in bone quality. Therefore, dialysis staff should pay attention to decreased physical activity in daily life to maintain BMD in patients undergoing HD.

Nutritional status might also be associated with the mechanisms between BMD and the PMI, because patients need to control various electrocytes for bone formation, including serum Ca, phosphate and Mg. However, they receive dietary intake restrictions including protein or vegetables, and therefore, nutrients required bone and muscle formation might be chronically in lacking in patients undergoing HD. In general, excessive Ca or phosphate could promote vascular calcification; therefore, serum Ca or phosphate must be managed appropriately based on CKD-MBD guidelines. Excessive Mg may lead to hypermagnesemia or fatal arrhythmia because patients cannot excrete Mg from the urine. However, Mg is one of the essential bone components, similarly to Ca or phosphate. Furthermore, vitamin K plays an important role in activating osteocalcin and promoting bone formation; however, in patients undergoing dialysis, vitamin K levels were reportedly lower than those in healthy participants^40^. As vitamin K is contained in seaweed or green and yellow vegetables, which also include high levels of potassium, vitamin K intake is restricted in dialysis patients. As these results, such intake restrictions of various foods might affect BMD and body size. Patients with low BMI or PMI might have low BMD, although management of electrocytes or intra-dialytic weight gain could be important. However, we did not investigate nutritional status in this study, and therefore, further studies are needed to clarify the association between BMD and PMI or BMI.

The present study has some limitations. First, the sample size was relatively small. Second, we measured the psoas muscle areas manually, not automatically using measurement software. However, we minimized the error due to evaluation by a radiologist. Third, we could not set a control group of healthy participants, because healthy participants or patients with non-dialysis CKD do not usually need to undergo both FN-BMD and CT scans at the same time. Therefore, this retrospective study did not collect control data. Finally, since this study was cross-sectional, we could not clarify whether PMI could affect FN-BMD in the medium to long-term in patients undergoing HD. Further studies are required to clarify the clinical implications and significance of the associations between PMI and BMD in the future.

In conclusion, FN-BMD was associated with sex and PMI in patients undergoing HD. Attention should be paid to the possibility of decreased BMD with decreased muscle mass.

## Data Availability

All data analyzed during this study are available within the paper.

## References

[1] Su, Y. C., Chang, S. F. & Tsai, H. C. The relationship between Sarcopenia and injury events: a systematic review and meta-analysis of 98,754 older adults. *J. Clin. Med.* **11**, 6474 (2022). 36362701 10.3390/jcm11216474 PMC9654071

[2] Suzuki, T. & Yoshida, H. Low bone mineral density at femoral neck is a predictor of increased mortality in elderly Japanese women. *Osteoporos. Int.* **21**, 71–79 (2010). 19499274 10.1007/s00198-009-0970-6

[3] Foley, R. N., Wang, C., Ishani, A., Collins, A. J. & Murray, A. M. Kidney function and sarcopenia in the United States general population: NHANES III. *Am. J. Nephrol.* **27**, 279–286 (2007). 17440263 10.1159/000101827

[4] Hanafusa, N. et al. Annual dialysis data report 2020, JSDT renal data registry. *Ren. Replace. Ther.* **10**, 14 (2024).

[5] Hamaguchi, Y. et al. Proposal for new diagnostic criteria for low skeletal muscle mass based on computed tomography imaging in Asian adults. *Nutrition* **32**, 1200–1205 (2016). 27292773 10.1016/j.nut.2016.04.003

[6] Okamura, H. et al. The impact of preoperative Sarcopenia, defined based on psoas muscle area, on long-term outcomes of heart valve surgery. *J. Thorac. Cardiovasc. Surg.* **157**, 1071–1079 (2019). 30139644 10.1016/j.jtcvs.2018.06.098

[7] Iwasaki, Y., Shiotsuka, J., Lefor, A. K. & Sanui, M. The psoas muscle index is associated with prognosis in elderly patients undergoing cardiovascular surgery. *Anesth. Pain Med.* **11**, e118608 (2021). 35075413 10.5812/aapm.118608 PMC8782061

[8] Kitamura, M. et al. The impact of muscle mass loss and deteriorating physical function on prognosis in patients receiving hemodialysis. *Sci. Rep.* **11**, 22290 (2021). 34785712 10.1038/s41598-021-01581-z PMC8595648

[9] Ito, K. et al. Muscle mass evaluation using psoas muscle mass index by computed tomography imaging in hemodialysis patients. *Clin. Nutr. ESPEN*. **44**, 410–414 (2021). 34330498 10.1016/j.clnesp.2021.04.029

[10] Takata, T. et al. Feasibility of computed tomography-based assessment of skeletal muscle mass in hemodialysis patients. *J. Nephrol.* **34**, 465–471 (2021). 32996109 10.1007/s40620-020-00871-5

[11] Senzaki, D. et al. Modeling low muscle mass screening in hemodialysis patients. *Nephron* **21**, 1–9 (2022). 10.1159/000526866 PMC10210080 36273447

[12] Hirata, M. et al. Factors affecting Psoas muscle Mass Index in patients undergoing peritoneal Dialysis. *Cureus* **16**, e56347 (2024). 38633934 10.7759/cureus.56347 PMC11021792

[13] Ketteler, M. et al. Diagnosis, evaluation, prevention, and treatment of chronic kidney disease-mineral and bone disorder: Synopsis of the kidney disease: improving global outcomes 2017 clinical practice guideline update. *Ann. Intern. Med.* **168**, 422–430 (2018). 29459980 10.7326/M17-2640

[14] Jørgensen, H. S., Lloret, M. J., Lalayiannis, A. D., Shroff, R. & Evenepoel, P. European renal osteodystrophy (EUROD) initiative of the CKD-MBD working group of the European Renal Association (ERA), and the CKD-MBD and Dialysis working groups of the European Society of Pediatric Nephrology. Ten tips on how to assess bone health in patients with chronic kidney disease. *Clin. Kidney J.* **17**, sfae093 (2024). 38817914 10.1093/ckj/sfae093 PMC11137676

[15] Miyakoshi, N., Hongo, M., Mizutani, Y. & Shimada, Y. Prevalence of Sarcopenia in Japanese women with osteopenia and osteoporosis. *J. Bone Min. Metab.* **31**, 556–561 (2013). 10.1007/s00774-013-0443-z 23515924

[16] Ito, K. et al. Skeletal muscle mass index is positively associated with bone mineral density in hemodialysis patients. *Front. Med. (Lausanne)*. **7**, 187 (2020). 32478086 10.3389/fmed.2020.00187 PMC7242614

[17] Kajiki, Y. et al. Psoas muscle index predicts osteoporosis and fracture risk in individuals with degenerative spinal disease. *Nutrition* **93**, 111428 (2022). 34474186 10.1016/j.nut.2021.111428

[18] Huang, C. B., Lin, D. D., Huang, J. Q. & Hu, W. Based on CT at the third lumbar spine level, the skeletal muscle index and psoas muscle index can predict osteoporosis. *BMC Musculoskelet. Disord*. **23**, 933 (2022). 36280811 10.1186/s12891-022-05887-5 PMC9590212

[19] Zhang, Y. et al. Correlation of psoas muscle index with fragility vertebral fracture: A retrospective cross-sectional study of middle-aged and elderly women. *Int. J. Endocrinol.* 4149468. (2022). (2022). 10.1155/2022/4149468 PMC9646299 36389125

[20] Assessment of fracture risk and its application to screening for postmenopausal osteoporosis. Report of a WHO Study Group. *World Health Organ. Tech. Rep. Ser.* **843**, 1–129 (1994). 7941614

[21] Tentori, F. et al. High rates of death and hospitalization follow bone fracture among hemodialysis patients. *Kidney Int.* **85**, 166–173 (2014). 23903367 10.1038/ki.2013.279 PMC3910091

[22] Wakasugi, M. et al. Increased risk of hip fracture among Japanese hemodialysis patients. *J. Bone Min. Metab.* **31**, 315–321 (2013). 10.1007/s00774-012-0411-z 23292163

[23] Barrera-Baena, P. et al. Serum phosphate is associated with increased risk of bone fragility fractures in hemodialysis patients. *Nephrol. Dial Transpl.* **39**, 618–626 (2023). 10.1093/ndt/gfad190 PMC10966329 37660283

[24] Sprague, S. M. et al. Diagnostic accuracy of bone turnover markers and bone histology in patients with CKD treated by dialysis. *Am. J. Kidney Dis.* **67**, 559–566 (2016). 26321176 10.1053/j.ajkd.2015.06.023

[25] Maruyama, Y. et al. A higher serum alkaline phosphatase is associated with the incidence of hip fracture and mortality among patients receiving hemodialysis in Japan. *Nephrol. Dial Transpl.* **29**, 1532–1538 (2014). 10.1093/ndt/gfu055 24642419

[26] Sakaguchi, Y., Hamano, T., Wada, A., Hoshino, J. & Masakane, I. Magnesium and risk of hip fracture among patients undergoing hemodialysis. *J. Am. Soc. Nephrol.* **29**, 991–999 (2018). 29191960 10.1681/ASN.2017080849 PMC5827604

[27] Paulin, T. K., Malmgren, L., McGuigan, F. E., Akesson, K. E. & Osteosarcopenia Prevalence and 10-year fracture and mortality risk - A longitudinal, population-based study of 75-year-old women. *Calcif Tissue Int.* **114**, 315–325 (2024). 38300303 10.1007/s00223-023-01181-1 PMC10957698

[28] Nakano, Y. et al. Effect of osteosarcopenia on longitudinal mortality risk and chronic kidney disease progression in older adults. *Bone* **179**, 116975 (2024). 37993037 10.1016/j.bone.2023.116975

[29] Yoshikoshi, S. et al. Prevalence of osteosarcopenia and its association with mortality and fractures among patients undergoing hemodialysis. *J. Bone Min. Metab.* **42**, 326–334 (2024). 10.1007/s00774-024-01503-9 38546869

[30] Kistler, B. M., Khubchandani, J., Jakubowicz, G., Wilund, K. & Sosnoff, J. Falls and fall-related injuries among US adults aged 65 or older with chronic kidney disease. *Prev. Chronic Dis.* **15**, E82 (2018). 29935079 10.5888/pcd15.170518 PMC6016407

[31] Kusunoki, H. et al. Relationships between cystatin C- and creatinine-based eGFR in Japanese rural community- dwelling older adults with Sarcopenia. *Clin. Exp. Nephrol.* **25**, 231–239 (2021). 33090338 10.1007/s10157-020-01981-x PMC7925493

[32] Naylor, K. L. et al. The three-year incidence of fracture in chronic kidney disease. *Kidney Int.* **86**, 810–818 (2014). 24429401 10.1038/ki.2013.547

[33] Noori, N. et al. Novel equations to estimate lean body mass in maintenance hemodialysis patients. *Am. J. Kidney Dis.* **57**, 130–139 (2011). 21184920 10.1053/j.ajkd.2010.10.003 PMC3026443

[34] Sen, E. I., Esmaeilzadeh, S. & Eskiyurt, N. Effects of whole-body vibration and high impact exercises on the bone metabolism and functional mobility in postmenopausal women. *J. Bone Min. Metab.* **38**, 392–404 (2020). 10.1007/s00774-019-01072-2 31897748

[35] Watanabe-Takano, H. et al. Mechanical load regulates bone growth via periosteal osteocrin. *Cell. Rep.* **36**, 109380 (2021). 34260913 10.1016/j.celrep.2021.109380

[36] Chopra, S., Morrow, M. M., Ngufor, C. & Fortune, E. Differences in physical activity and sedentary behavior patterns of postmenopausal women with normal vs. low total hip bone mineral density. *Front. Sports Act. Living*. **2**, 83 (2020). 33345074 10.3389/fspor.2020.00083 PMC7739614

[37] Young, H. M. L. et al. Standardizing the measurement of physical activity in people receiving haemodialysis: considerations for research and practice. *BMC Nephrol.* **20**, 450 (2019). 31801480 10.1186/s12882-019-1634-1 PMC6894215

[38] Choi, S. M., Cho, S. H. & Kim, B. C. Association between freezing of gait and bone mineral density in patients with Parkinson’s disease. *Neurol. Sci.* **42**, 2921–2925 (2021). 33230756 10.1007/s10072-020-04920-6

[39] Nagata, J. M. et al. Associations between exercise, bone mineral density, and body composition in adolescents with anorexia nervosa. *Eat. Weight Disord*. **24**, 939–945 (2019). 29949128 10.1007/s40519-018-0521-2 PMC6286679

[40] Mizuiri, S. et al. Relationship of matrix Gla protein and vitamin K with vascular calcification in hemodialysis patients. *Ren. Fail.* **41**, 770–777 (2019). 31538831 10.1080/0886022X.2019.1650065 PMC7011966

